# 2D-DIGE-Based Proteomic Profiling with Validations Identifies Vimentin as a Secretory Biomarker Useful for Early Detection and Poor Prognosis in Oral Cancers

**DOI:** 10.1155/2022/4215097

**Published:** 2022-04-22

**Authors:** Ananthi Sivagnanam, Vidyarani Shyamsundar, Pallavi Kesavan, Arvind Krishnamurthy, Soundara Viveka Thangaraj, Divyambika Catakapatri Venugopal, Hemashree Kasirajan, Pratibha Ramani, Vinutha Rachapudi Sarma, Vijayalakshmi Ramshankar

**Affiliations:** ^1^Department of Preventive Oncology (Research), Cancer Institute (WIA), Adyar, Chennai, 600020 Tamil Nadu, India; ^2^Centre for Oral Cancer Prevention and Research, Sree Balaji Dental College and Hospital, Pallikaranai, Chennai, 600100 Tamil Nadu, India; ^3^Department of Surgical Oncology, Cancer Institute (WIA), Adyar, Chennai, 600020 Tamil Nadu, India; ^4^Department of Oral Medicine and Radiology, Sri Ramachandra Institute of Higher Education and Research (DU), Porur, 600016, Chennai, Tamil Nadu, India; ^5^Department of Oral Pathology and Maxillofacial Pathology, Saveetha Dental College, Velappanchavadi, 600077, Chennai, Tamil Nadu, India; ^6^Public Health Foundation of India, New Delhi 110017, India

## Abstract

Oral tongue squamous cell carcinoma (OTSCC) is an aggressive cancer with high morbidity and mortality rates, despite multimodality management. There are currently no clinically relevant molecular markers that identify patients at higher risk of recurrence and failure. We undertook 2D-DIGE proteomic profiling to study the differentially expressed proteins in OTSCC evaluating their role in prognosis. 2D-DIGE coupled with tandem mass spectrometry was performed on tissues obtained from early staged OTSCC along with its paired apparently adjacent normal tissue samples (*n* = 10). Top upregulated protein was validated using immunohistochemistry (*n* = 345), comprising of retrospective early stage OTSCC (*n* = 150) and prospective series of oral precancers, normal, and oral cancers (*n* = 195). Saliva samples collected from oral cancer and precancer samples were analyzed by ELISA (*n* = 146). We found statistically significant differential expression in 151 proteins out of 700 proteins quantified. Top ten differentially regulated proteins were identified using mass spectrometry analysis. We found vimentin, the mesenchymal protein, to be the most upregulated protein in tongue tumor tissues compared to adjacent apparent normal tissues. Vimentin was found to be significantly overexpressed in oral precancers along with cancers compared to normal tissues. The vimentin expression correlated significantly with differentiated states of oral precancers and cancers. Vimentin was also detected at significantly higher levels in saliva collected from oral precancer and cancer patients compared to normal healthy volunteers. Validation of vimentin in an independent series of retrospective early staged OTSCC showed that the vimentin expression is significantly associated with treatment failures and poorer DFS. The vimentin expression is useful as both poor prognostic and early detection marker in oral cancer. Vimentin detection in saliva can be a diagnostic test to detect oral precancers that may have malignant potential, needing closer follow-up, and disease monitoring.

## 1. Introduction

Oral tongue squamous cell carcinoma (OTSCC) represents a major portion of oral cavity cancers, especially in India. Studies show a sharp increase in the incidence of tongue cancers in India [1]. Our earlier studies on south Indian patient population have shown that early staged tongue cancers (T1 and T2) constitute nearly 45% of all OTSCCs [2]. Studies have shown that OTSCC occurs at a younger age than cancers occurring in other subsites of the oral cavity [3]. Despite being detected at an early stage, about 40% of patients still die of the disease and need tailored treatment. Depth of invasion, tumor grade, and perineural invasion are some of the factors indicating an aggressive phenotype but till date, there are no relevant molecular markers indicating the high-risk tumors.

Proteomics helps to study the complete protein complements of the cell, which is a promising approach for the identification of novel protein biomarkers. These proteins can be used as key targets for therapeutic intervention and also as promising candidates for early detection of cancers [4, 5]. We have some recent studies showing the preliminary application of proteomics for the identification of biomarkers for oral squamous cell carcinoma (OSCC) [6, 7]. Comparison of protein expression profiles between OSCC and normal cell lines or tissues has revealed replicable and significant changes in the expression levels of number of proteins, including some metabolic enzymes, modulators of signal transduction pathways, and oncoproteins [8]. In the current study, we performed two-dimensional difference gel electrophoresis- (2D-DIGE-) based proteomic profiling coupled with mass spectrometry approach and validated the expression of top upregulated protein vimentin, eventually to explore the prognostic stratification of early staged OTSCC. We additionally studied the vimentin expression in oral precancers and cancers compared with normal tissues. ELISA for vimentin was attempted to evaluate the secretion of vimentin in saliva samples from normal healthy volunteers compared with patients presenting oral leukoplakia, oral submucous fibrosis (OSMF), and OSCC.

## 2. Materials and Methods

### 2.1. Patients and Tissue Specimens

All research involving human participants had been approved by the authors' Institutional Review Board (IRB), and all clinical investigations were conducted according to the principles expressed in the Declaration of Helsinki. A written informed consent was obtained from all the participants, and the content of the informed consent was approved by the respective Institutional Research Board, namely, Cancer Institute WIA; Protocol 1 HNCOG (Cancer Institute, Women India Association; Protocol 1 Head and Neck Cooperative Oncology Group), SBDCECM105/13/58 (Sree Balaji Dental College and Hospital Ethical Committee Meeting reference number 105/13/158), and the Department of Oral Medicine and Radiology, Sree Ramachandra Dental College and Hospital from June 2018 till January 2019. Institutional Ethics Committee approval (IEC No.CSP/17/AUG/60/239) and Ethics approval from Department of Oral and Maxillofacial Pathology, Saveetha Dental College and Hospital from December 2017 to August 2019 (SRB/SDMDS11/170MP/01) was obtained before the commencement of the study.

### 2.2. Patient Tissue Samples

Histologically apparent adjacent normal tongue tissues along with paired early staged OTSCC tumor tissues (*n* = 10) were obtained from patients presenting OTSCC and undergoing surgery. Formalin fixed paraffin embedded (FFPE) samples from buccal leukoplakia (*n* = 50) and oral cancers (*n* = 71) were obtained from Sree Balaji Dental College and Hospital and formalin fixed tissues from OSMF samples (*n* = 32), and normal buccal mucosa tissues (*n* = 42) were obtained during third molar extraction and were collected from Sree Ramachandra Dental College and Hospital. This was an independent cohort (*n* = 195) for validation studies. Additionally, FFPE sections from retrospective series of exclusively early staged OTSCC patients (T1 and T2) (*n* = 150) were obtained from Cancer Institute WIA who had been treated between 1995 and 2000 for validation studies of the findings with the complete treatment follow-up. All the FFPE sections (*n* = 345) used for the study were histologically examined by oral pathologist VS and PR.

### 2.3. Patient Saliva Samples

ELISA was done for saliva samples (*n* = 146) collected from oral cancer patients (*n* = 60), patients presenting oral potentially malignant lesions (*n* = 47) and absolute normal volunteers (*n* = 39). The samples were obtained from Saveetha Dental College and Hospital and Sree Ramachandra Dental College and Hospital. The study participants were requested to refrain from drinking, eating, chewing tobacco, or smoking 1 hour prior to the collection of saliva. After obtaining the informed consent of the patient, 0.5 to 1 ml of whole unstimulated saliva was collected by passive expectoration, and patients were asked to spit into a 50 ml sterile tube containing 10 *μ*L of proteinase inhibitor (Proteinase inhibitor cocktail P2714 Sigma-Aldrich). The saliva samples were transferred to 1.5 ml sterile microtubes and centrifuged for 3 minutes at 13,000 rpm. Supernatants, separated from the cellular phase, were immediately aliquoted and stored at -80°C within 60 minutes after saliva collection.

### 2.4. Proteomics-Proteins Labeling with CyDyes

Pooled OTSCC tumor and pooled adjacent uninvolved tissues were used for the proteomic profiling. Lysine labeling protocol (minimal labeling) used in this study is described previously [9]. The processed tissue proteins were labeled individually with dyes Cy3 and Cy5 while the pooled tissue proteins prepared by mixing equal aliquot of protein from all samples in an experimental set up were labeled with Cy2. The final volume for all preparations was adjusted to a total of 340 *μ*L with rehydration buffer (7 M urea, 2 M thiourea, 1% IPG buffer, 50 mM DTT, 4% CHAPS, and a trace amount of bromophenol blue). A reciprocal labeling experiment was also performed.

### 2.5. Proteomics–2D-DIGE

2D-DIGE of CyDye labeled proteins was done as described before [10, 11] with the following modifications. Eighteen cm IPG strips of pH 4–7 (GE Healthcare, Uppsala, Sweden) were employed in the first dimension. Labeled proteins were focused for a total of 80000 Vhs at a constant temperature (20*μ*C) under linear voltage ramp after an active IPG rehydration at 30 V in a IPGPhor III (GE Healthcare, Uppsala, Sweden) apparatus. Following IEF, each IPG strip was placed in the equilibration buffer containing 2% DTT first followed by incubation in another buffer in which the DTT was replaced by 2.5% iodoacetamide. The second dimension PAGE (12.5%) was carried out in EttanDaltSix systems (GE Healthcare, Uppsala, Sweden) at 1 W/gel for 1 hr and 13 W/gel for 5 hr. All experimental procedures were performed in dim light or in the dark.

### 2.6. Protein Visualization and DeCyder image analysis

The protocols for the protein visualization and image analysis using DeCyder been mentioned previously [12]. Briefly, after second dimension electrophoresis, the gels were scanned with Typhoon FLA 9500 Variable Mode Imager (GE Healthcare, Uppsala, Sweden). Cy2, Cy3, and Cy5 images were captured using the settings recommended by the manufacturer. A DeCyder differential in-gel analysis (DIA) module was used for the image analysis between samples within the same gel while a DeCyder biological variation analysis (BVA) module was performed for pairwise image analysis among multiple gels. Student's *t*-test and ANOVA were used to compare the average spot volume and differences of protein abundance for all detectable spots between the tumor and normal groups. Reciprocal dye labeling was performed to normalize bias in labeling.

### 2.7. Protein Identification and Mass Spectrometry (MS)

Pooled tongue tissue proteins (250 *μ*g) were separated on 18 cm IPG strips of pH 4-7 in the first dimension. First and second dimension electrophoresis were done as given under the 2D-DIGE method. The second dimension gels were stained with colloidal Coomassie blue G-250, gel spots from this preparative gel were excised manually for in-gel trypsin digestion, and LC-MS/MS was performed. Extracted peptides were dried under vacuum for 90 min and stored at 4°C. Zip tip purified peptides were analyzed using nano-RPLC (Thermo Scientific, USA) coupled with an Orbitrap Elite Mass spectrometer (Thermo Scientific, USA). Peptides were ionized by positive mode electrospray with an ion spray voltage of 1.9 kV. The MS data were acquired in positive ion mode over mass range m/z 350–4000 Da using Xcalibur software (version 2.2.SP1.48) (Thermo Scientific USA). MS data were analyzed using Proteome Discoverer software v.1.4 (Thermo Scientific) using the SEQUEST algorithm with database downloaded from Uniprot as described earlier [9]. The combined list of official gene symbols corresponding to the identified proteins was used for input. We used STRING (http://www.string.db.org/) [13] for network construction.

### 2.8. Immunohistochemistry (IHC)

The IHC detection methods were as mentioned previously [14]. Briefly, IHC for vimentin was performed on 5 *μ*m sections of FFPE tissues. The sections were deparaffinized in xylene and rehydrated in absolute ethanol. Antigen retrieval was done with 0.05 M citrate Buffer (pH 9) in pressure cooker for 20 minutes. Endogenous peroxidase activity was blocked by incubation in 0.03% hydrogen peroxide in distilled water for 10 minutes and then washed with phosphate buffered saline (PBS). Sections were counterstained with hematoxylin, dehydrated, and mounted in DPX. Positive controls and negative controls were included appropriately where primary antibody was replaced with 2% BSA in negative control. Immunostaining of the sections was reviewed with the corresponding hematoxylin and eosin-stained sections.

### 2.9. IHC Scoring

Immunohistochemical scoring for the target was done as described earlier [15]. Briefly, the percentage grade of stained tumor cells was scored as 0, negative; 1, <10%; 2, 11–50%; 3, 51–80%; or 4, >80% positive cells, and the intensity of stain was scored as 0, negative; 1, weak; 2, moderate; or 3, strong. The immunoreactive (IR) score was calculated as a product of the percentage grade and intensity score with the IR score ranged from 0 to 12. The immunoreactivity was divided into three groups based on the final score: negative immunoreactivity was defined as a total score of 0, low immunoreactivity was defined as a total score of 1–4, and high immunoreactivity was defined as a total score > 4. The immunostaining of the tumor invasive front was evaluated using the same method as mentioned for tumor areas.

### 2.10. ELISA in Saliva Samples

The RayBio® Human Vimentin ELISA (Enzyme-Linked Immunosorbent Assay) kit is used to quantify the expression of vimentin in saliva samples (*n* = 146), of which saliva samples from patients with oral cancer (*n* = 60), patients with oral potential premalignant lesions (*n* = 47), and healthy volunteers (*n* = 39) were used. This is an in vitro enzyme-linked immunosorbent assay for the quantitative measurement of human vimentin in saliva samples. This assay employs an antibody specific for human vimentin coated on a 96-well plate. Standards and samples are pipetted into the wells, and vimentin present in a sample is bound to the wells by the immobilized antibody. The wells are washed and biotinylated anti-human vimentin antibody is added. After washing away unbound biotinylated antibody, HRP-conjugated streptavidin is pipetted to the wells. The wells are again washed, a TMB substrate solution is added to the wells, and color develops in proportion to the amount of vimentin bound. The Stop Solution changes the color from blue to yellow, and the intensity of the color is measured at 450 nm. The standard graph was plotted with the vimentin standard protein provided in the kit. Using the standard graph, the protein concentrations were extrapolated for the unknown OD values obtained from saliva samples obtained from patients and healthy volunteers.

### 2.11. Statistical Analysis

The relative levels of stained protein spots compared with the internal standard spots were analyzed by DeCyder Difference In-gel Analysis (DIA) and DeCyder Biological Variation Analysis (BVA) software modules (GE Healthcare). Student's *t*-test was used to calculate statistically significant differences between 2 groups in relative abundance of individual protein spots among the groups in 2D-DIGE. *p* < 0.05 was considered statistically significant. Other statistical analysis was done using SPSS (IBM Corporation version 16).

## 3. Results

### 3.1. 2D-DIGE and Mass Spectrometry Analysis

**Figure 1:**
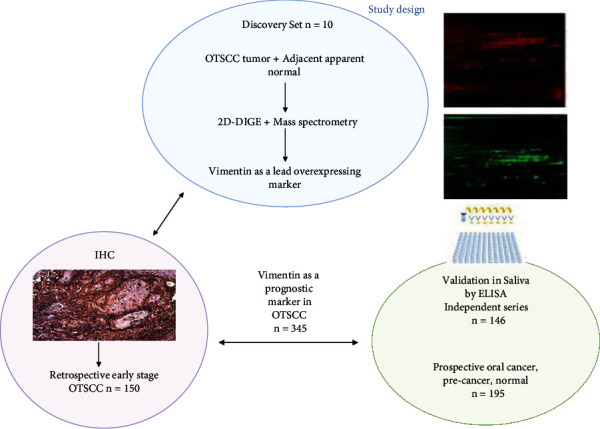
Study design for vimentin as prognostic marker in OTSCC.

**Figure 2:**
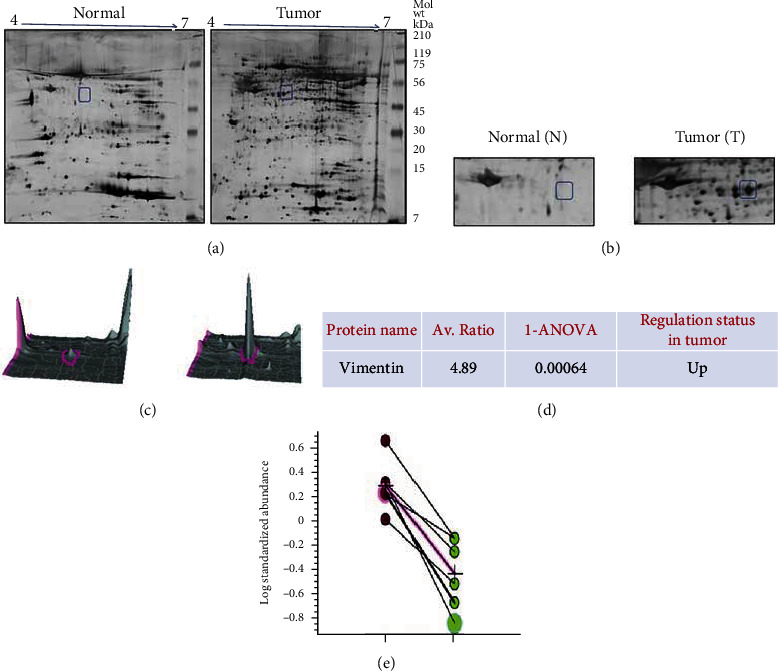
(a, b) Representative 2DE gels of normal and tumor sample. (c) 3D view and graphical log value representation of the vimentin protein spot expression in normals and tumor sample. (d) One-way ANOVA *p* value test for statistical analysis. (e) Fold ratio analysis for differentially regulated proteins.

Figure 1 shows the study design with the number of samples used in the discovery phase and validation phase. Comparative proteomic analysis of pooled tongue tissue samples obtained from OTSCC patients compared to pooled adjacent apparent normal samples is shown in Figure 2. More than 95 protein pairs were obtained in the image analysis platform, among which 45 were upregulated in tumor samples compared with adjacent normal protein samples. Out of the differentially expressed proteins, top 10 differentially expressed spots were taken for mass spectrometry.  Table 1 describes the protein identification details for the differentially regulated proteins with the accession number matched in the database, mass spectrometry probability score, and percentage of sequence coverage match.  Table 2 describes the average fold ratio with ANOVA value for differentially regulated proteins. The top 10 differentially regulated proteins have been listed in Table 2, in which eight proteins were significantly upregulated, and two proteins were significantly downregulated in tumor samples. Quantitative 2D-DIGE proteomic approach coupled with tandem mass spectrometry identified vimentin as the topmost upregulated protein in OTSCC (Figure 2 (a)).

Figure 2(b) shows the highly upregulated protein vimentin marked in a box region across both the gels. The 3D view and the graphical log value of the vimentin protein spot expression in 2D-DIGE gel were analyzed and represented in Figure 2(c) along with the magnified 2D gel view. The average ratio of upregulation of vimentin was 4.89-fold higher compared to the normal counterpart. The one-way ANOVA *p* value was found to be 0.00064 (Figure 2(d)). The log standardized abundance was significantly higher compared to the normal tissue protein (Figure 2(e)).

### 3.2. Functional Classification of the Identified Vimentin Protein and Biological Network Analysis

**Figure 3:**
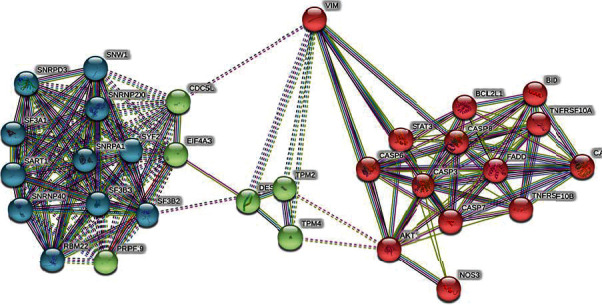
Cluster analysis for the differentially regulated protein vimentin using STRING database.

The STRING [13] cluster analysis revealed that vimentin forms a strong protein interactions with other partners, comprising of three major networks: first cluster is with most of the small nuclear ribonucleoproteins (snRNPs), second cluster with different members of tropomyosin, and third cluster with many caspases (Figure 3). All these protein networks are known to have a key role in different tumorigenesis pathways; thus, this interaction analysis describes the significance of the identified vimentin protein's regulation in tumorigenesis process.

### 3.3. Validation of the Vimentin Expression to Evaluate the Role in Early Staged OTSCC

To validate the expression of vimentin and its role in OTSCC, we undertook a retrospective cohort of exclusively early staged (T1 and T2) OTSCC patients (*n* = 150) treated as per the decision of the multispecialty board between 1995 and 2007. The clinicopathological features of early staged OTSCCs analyzed based on the vimentin protein expression are shown in Table 3. Median age of the cohort was 55 years, median OS was 74 months, and DFS was 22 months. The pattern of the vimentin expression was predominantly cytoplasmic. Positive immunoreactivity for vimentin was identified in 58 (38.6%) patients, and vimentin was negative in 92 (61.31%) patients.

### 3.4. Expression of Vimentin in Early Staged OTSCC

Among the vimentin positive tumors, 46.8% (44/94) belonged to stage 2 compared to 25% (14/56) in stage 1 which was (*p* = 0.008; *χ*^2^ = 7.038). Among the vimentin positive tumors, 48.3% (43/89) had a tumor size > 2 cm compared to 25.9% (15/58) of vimentin positive immune-expression in tumors of size less than 2 cm which was (*p* = 0.009; *χ*^2^ = 9.394). There was decreased vimentin positivity identified in tongue tumors that was ulcerated 10% (2/20) compared to the exophytic and infiltrating (*p* = 0.014; *χ*^2^ = 8.536).

The patients showing strong and positive immunoexpression of vimentin had a higher failure rates (45.9% (39/85) vs. 29.2% (19/65)) which was statistically significant, (*p* = 0.038; *χ*^2^ = 4.306), and the data is represented in Table 3. Positive immunoexpression of vimentin was significantly associated with locoregional recurrence (53.8% (21/39) vs. 29.2% (19/65)) patients showing no evidence of disease which was (*p* = 0.028; *χ*^2^ = 10.892).

Vimentin at ITF was positive in 47.6% (50/105) of patients whose tumors showed an invasive tumor front. Among the patients who showed locoregional recurrence with ITF in the tumors, 68% (17/25) showed a positive vimentin at ITF compared to 32% (8/25) patients whose tumors ITF had a negative immune expression as shown in Table 4.

### 3.5. Patients Survival among the Early Staged OTSCC

**Figure 4:**
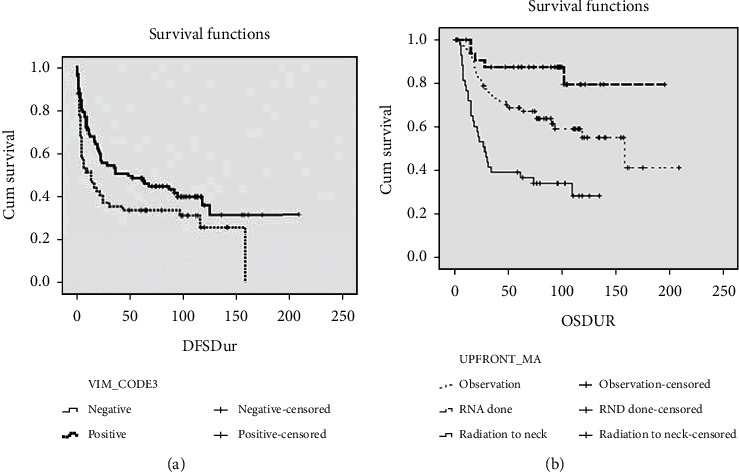
Kaplan Meier survival plots of oral cancer patients: (a) Kaplan Meier curves showing survival fractions in patients with vimentin expression pattern and (b) Kaplan Meier curves showing survival fractions in patients undergoing the different types of upfront neck management.

Patients whose tumors had positive vimentin expression had decreased DFS compared to the patients whose tumors had negative vimentin expression, and this association was statistically significant (log rank = 4.068; *p* = 0.044) (Figure 4(a)). The patients in this cohort were subjected to upfront neck management, namely, neck node observation (*n* = 73), modified radical neck dissection (MRND) (*n* = 33), and radiation to neck (*n* = 44). Kaplan Meier survival curves of DFS based on upfront neck management showed that patients who underwent MRND have a better survival (*p* = 0.006; log rank = 10.094) compared to patients given radiation to neck (Figure 4(b)). The patients undergoing modified neck dissection for neck management had the best survival among the early staged OTSCC.

### 3.6. Correlation of the Vimentin Expression with Dysplasia in Oral Precancers

Based on the findings of the retrospective study, we undertook another cohort of patients (*n* = 196) with oral leukoplakia (*n* = 50), OSMF (*n* = 32), and invasive cancers involving buccal cavity (to ascertain if vimentin can be useful in oral cancer inclusive of both sites oral tongue and buccal cavity. This cohort had 82 oral precancers comprising of leukoplakia (*n* = 50) and OSMF (*n* = 32) along with normals (*n* = 42) and cancers of buccal cavity (*n* = 72).  Table 5 shows the demographic details of subjects and patients who presented with oral precancers at different stages and oral cancers presenting to the dental clinic. The median age of this group was 45 years.

**Figure 5:**
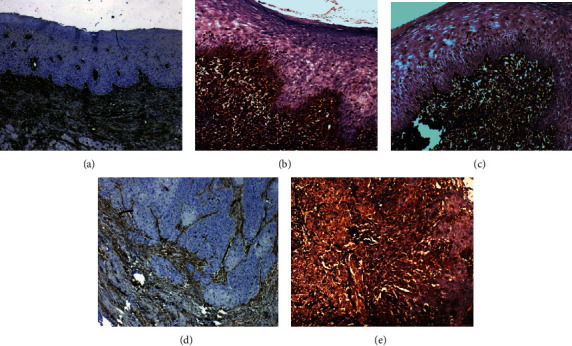
IHC analysis for the vimentin protein expression. (a) Normal oral epithelium with vimentin positive only in the connective tissue. IHC, ×20, (b) mild dysplastic oral mucosa showing vimentin positivity in the basal epithelial cells and vimentin positive connective tissue. IHC, ×20, (c) mild dysplastic oral mucosa with vimentin negative epithelium and positive connective tissue. IHC, ×20, (d) well differentiated squamous cell carcinoma with vimentin negative tumor cells and vimentin positive surrounding connective tissue. IHC, ×20, (e) moderately differentiated squamous cell carcinoma with vimentin positive tumor cells and vimentin positive surrounding connective tissue. IHC, ×20.

The vimentin expression was analyzed by IHC in totally 195 samples, of which 144 samples (73.9%) showed the vimentin negative expression and 51 samples (26%) showed the vimentin positive expression in IHC analysis. The median age of this cohort was 45 years and age had a significant association with the vimentin positive expression (*p* ≤ 0.001; *χ*^2^ = 16.163). The immunoexpression pattern of vimentin is represented in Table 5 describing the positive expression of vimentin having a significant association (*p* ≤ 0.001: *χ*^2^ = 68.524) with oral precancers and cancers compared to apparent normals. Interestingly, vimentin was significantly correlated to the dedifferentiated state of the oral precancers with oral cancers (*p* ≤ 0.001; *χ*^2^ = 77.037). Vimentin is a significant biomarker for oral precancers that may have an aggressive potential to turn into malignancy.  Figure 5 shows the IHC-based patterns of the vimentin expression in oral cancers.

### 3.7. Vimentin Secretion in Saliva Samples

**Figure 6:**
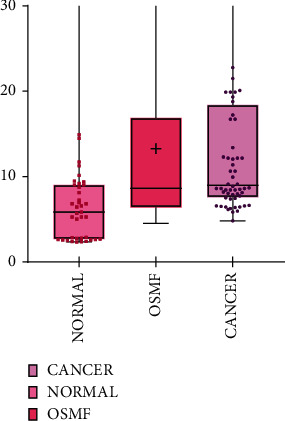
Scatter plot representation for vimentin ELISA with data summary values.

**Figure 7:**
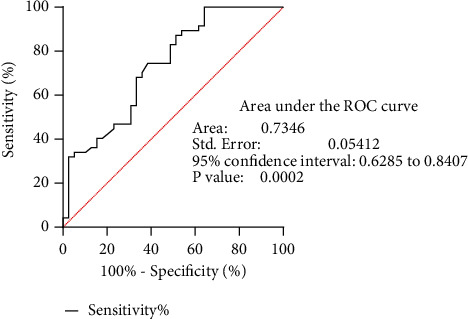
ROC curve and AUC analysis for Vimentin ELISA.

Since tissue availability in oral cancers and precancers involve invasive procedures as a biopsy, we wanted to evaluate if vimentin could be detected in saliva, as saliva can serve as a noninvasive medium for the early detection and also for disease monitoring. Vimentin secretion in saliva could be detected as significantly high in oral cancer and precancer patients. The concentration obtained in salivary ELISA for vimentin detection range from 2.33 to 3.34 ng/ml for healthy volunteer samples; for precancer samples, the concentration ranges from 4.5 to 5.02 ng/ml and for cancer samples, the concentration ranges from 4.82 to 1903.9 ng/ml. The fold increase between healthy volunteer samples and precancer samples was statistically significant. The differential expression was analyzed using SPSS software and observed to be statistically significant, and the data is represented in Figure 6. The diagnostic potential of the underlying pathological implication could be detected by ROC curve analysis showing AUC = 0.735 which had a high statistical significance (*p* ≤ 0.001) (Figure 7).

## 4. Discussion

Vimentin is a well-known mesenchymal protein acting as a scaffold for signaling proteins that are important for cancer cell invasion [16] wound healing, tissue repair [17] tissue ageing, and apoptosis [18, 19]. This study describes the role of vimentin that emerged as the most upregulated protein in OTSCC by quantitative proteomics. We have validated the expression of vimentin in early staged OTSCC inferring its role as a poor prognostic indicator. The role of vimentin was further evaluated in oral precancers and in saliva indicating that vimentin can be a very useful marker in oral cancer for determining prognosis and can be used for early detection of the disease in saliva and oral precancer tissues. Vimentin can be good biomarker because none of the normal buccal mucosa tissues expressed vimentin as expression of vimentin is indicative of mesenchymal transition. The current study showed the aberrant vimentin expression in oral premalignant lesions and oral cancers.

Previous study done as a meta-analysis of differentially expressed genes in OTSCCs showed the comprehensive expression profiling of genes identifying the role of extracellular matrix with EMT-based deregulation in OTSCC showing the role of tumor microenvironment in OTSCC with a number of extracellular matrix (ECM) components playing a crucial role in patient prognosis [14].

As the first step, we evaluated the global proteomic profiles in early staged oral tongue cancer samples by 2D-DIGE followed by mass spectrometry analysis. 2D-DIGE technique enables direct comparison of protein profile between tumor and normal samples on the same single 2D gel, thus reducing technical variability which could affect the expression pattern of proteins. Recent studies have used the conventional 2D electrophoresis [7] and identified a panel of 12 proteins in tongue cancer, but the absence of validations in normal tissues in the same gels can possibly lead to biased conclusions owing gel-to gel variations. To overcome this, we attempted 2D-DIGE based proteomic discovery in the current study and showed that proteins involved in cytoskeletal remodeling are involved in the process of tumorigenesis. Proteomics approach can give rise to several markers, as shown in the current study but these biomarkers need to be chosen as per their clinical relevance based on validation studies. Our studies showed an additional 9 top differentially regulated proteins that can be subsequently validated in oral cancer tissue and saliva samples. Along with vimentin, we also obtained laminin A/C transcript, myoglobin as significant markers by proteomics approach, and they are well known stromal components playing an important role in ECM modulation. Vimentin has been evaluated as a useful marker for aggressive pathology and poor prognosis in tongue cancers, and its utility can be explored to assess patients who are more likely to fail treatment, despite being early staged.

To validate this finding, as a second step, we evaluated the vimentin expression in retrospective series of exclusively early staged OTSCC. Early staged OTSCC needs a biomarker to identify the patients who are more likely to fail despite being in T1 or T2 stage. The aggressive nature of OTSCC is reflected by the increased rates of local recurrence, occult node, and distant metastasis. Though several histological features like extracapsular spread, perineural invasion, and presence of lymphovascular emboli are adverse factors, there is still an unanswered need of objective molecular markers that can be useful to identify patients needing further attention.

This study showed that upfront neck management was an important factor to predict event free survival. Patients who underwent modified neck dissection had the best overall survival among the early staged OTSCC showing the importance of neck dissection in OTSCC. This result was in agreement to the previous randomized controlled trial where elective neck dissection showed higher rates of overall and disease-free survival in early staged OTSCC. [20] We did not find a significant correlation with the overall survival and vimentin expression unlike the disease-free survival which is similar to previous reports [21]. The positive vimentin expression was found to be associated with increased stage, size of the tumor, treatment failures, locoregional recurrence, and poorer disease-free survival. Earlier studies have identified the vimentin overexpression to be a poor prognostic indicator in OTSCC by univariate analysis [21–24]. Our study confirms the earlier findings. It has been suggested that cancer cells present in the invasive tumor front (ITF) are more aggressive in terms of their metastatic potential [25]. Our study emphasizes that the expression of vimentin assessed at ITF can indicate the EMT switch which is known to be associated with increased motility and invasiveness. We found a significant association with locoregional recurrence and vimentin expression at ITF. Vimentin at ITF and tumor sites has been shown to be strongly correlated to aggressive phenotype contributing to poor prognosis [26]. The aberrant expression of vimentin incriminated in various epithelial cancers [27–32] including OSCC [33]. Vimentin has been shown as a predictive biomarker for tumor growth and metastasis, although its understanding is limited in OTSCC prognosis [34, 35].

As a third step, we wanted to evaluate the significance of vimentin in oral premalignant lesions that would comprise of leukoplakia, OSMF as well as buccal cancers. We wanted to evaluate if vimentin can be a biomarker for oral cancers, comprising of both the major sites buccal as well as tongue. We found a significant association of the vimentin expression in oral precancers and cancers. Increased age was correlated to the vimentin expression mainly because of higher incidence of cancers in subjects with increasing age. Most of the potentially malignant disorders are asymptomatic, and treatment can be of three types, namely, close observation, surgical excision/ablation, and medical treatment. There is a lack of standardized diagnostic criteria in visual inspection of oral cavity to identify potentially malignant lesions that may eventually progress. Previous studies have shown that vimentin expression in lesions of leukoplakia and submucous fibrosis could be an early event in tobacco and areca nut associated tumorigenesis process.

As the fourth step, we have evaluated the vimentin expression in saliva as a noninvasive means and have shown that vimentin can be a good marker for both early detection and disease monitoring in oral cancers. Our current study confirms this finding with a higher vimentin expression in saliva samples using the ELISA method with a significant diagnostic potential for identifying patients with poor prognosis. This can be validated in higher number of samples of oral precancers and cancers for early detection and disease monitoring. To our knowledge, this is the first study evaluating vimentin in saliva from our country. Vimentin has been shown to be secreted by distinct population of vascular endothelial cells and activated macrophages and can accumulate in the blood previously [36, 37].

Vimentin secretion has been shown to be induced by proinflammatory cytokines TNF-*α*, and LPS suggesting that vimentin secretion is an inflammatory response [38, 39]. We found that vimentin secretion was indeed very elevated in certain oral precancer samples that they could be an inflammatory response and more prone for malignant transformation in future.

A recent report reviewing all the promising biomarkers identified in tongue cancers have shown vimentin as one of the strongest biomarker with significant relevance as a marker with clinical utility [40] proving that it is an important marker of OTSCC which innately shows a higher propensity to metastasize confirming our reports. As per our findings, vimentin in precancers can help identify patients who are most likely to progress to malignancy. As a result, it can be a useful marker for early detection. Up to our knowledge, this is the first report to describe the status of the vimentin expression in saliva samples from precancer and cancer patients with oral squamous cell carcinoma. Oral cancer is a very common cancer in the Indian population, and there were very few studies that have aimed to identify biomarkers with a validation study. We have identified the candidate proteins altered in the Indian population and validated them with both IHC and ELISA analysis.

## 5. Conclusions

In conclusion, 2D-DIGE coupled with tandem mass spectrometry was found useful to identify differentially expressed proteins in OTSCC tissues. All the quantitative tissue proteomic-based markers identified in current study need validation in OTSCC tissues as a prospective study with larger numbers of samples. The current study has been pursued for vimentin, a well-characterized EMT marker. It was found clinically relevant to prognosticate early OTSCC patients most likely to fail treatment, requiring specific tailored treatment. Vimentin was also useful as an early detection biomarker of precancers in oral cavity. In addition, the vimentin protein expression has been validated in saliva samples obtained from precancer and cancer patients and found to be significantly upregulated when compared to normal samples, proving its role as a useful biomarker for the early detection and disease monitoring.

## Figures and Tables

**Table 1:** List of differentially regulated proteins identified using mass spectrometry.

Spot ID	Accession	Description	Score	Coverage	# proteins	# unique peptides	# peptides	# PSMs	# AAs	MW [kDa]	Calc. pI
1	B0YJC4	Vimentin OS = Homo sapiens, GN = VIM, PE = 3, SV = 1 − [B0YJC4_HUMAN]	76.06	51.97	31	12	28	52	431	49.6	5.25
2	P60174	Triosephosphate isomerase OS = Homo sapiens, GN = TPI1, PE = 1, SV = 3 − [TPIS_HUMAN]	52.41	49.30	3	2	13	29	286	30.8	5.92
3	P30041	Peroxiredoxin − 6 OS = Homo sapiens, GN = PRDX6, PE = 1, SV = 3 − [PRDX6_HUMAN]	49.05	51.79	3	9	13	31	224	25.0	6.38
4	P30084	Enoyl − CoA hydratase, mitochondrial OS = Homo sapiens, GN = ECHS1, PE = 1, SV = 4 − [ECHM_HUMAN]	63.59	45.86	1	9	15	39	290	31.4	8.07
5	P52565	Rho GDP − dissociation inhibitor 1 OS = Homo sapiens GN = ARHGDIA PE = 1, SV = 3 − [GDIR1_HUMAN]	135.67	57.35	8	10	20	138	204	23.2	5.11
6	Q32Q12	Nucleoside diphosphate kinase OS = Homo sapiens, GN = NME1 − NME2, PE = 2, SV = 1 − [Q32Q12_HUMAN]	43.60	47.60	9	4	9	21	292	32.6	8.48
7	P02671	Fibrinogen alpha chain OS = Homo sapiens, GN = FGA, PE = 1, SV = 2 − [FIBA_HUMAN]	42.42	32.68	4	5	22	40	866	94.9	6.01
8	Q5I6Y6	Lamin A/C transcript variant 1 OS = Homo sapiens, GN = LMN, A PE = 2, SV = 1 − [Q5I6Y6_HUMAN]	29.77	20.18	9	6	11	17	664	74.0	7.18
9	P02144	Myoglobin OS = Homo sapiens, GN = MB, PE = 1, SV = 2 − [MYG_HUMAN]	81.93	68.83	8	8	16	50	154	17.2	7.68
10	P24844	Myosin regulatory light polypeptide 9 OS = Homo sapiens, GN = MYL9, PE = 1, SV = 4 − [MYL9_HUMAN]	51.33	54.65	9	7	9	25	172	19.8	4.92

**Table 2:** List of differentially expressed proteins with regulation status, fold ratio, and ANOVA.

Spot ID	Gene ID	Regulation status in tumor	Average fold ratio	ANOVA
1	VIM	UP	4.89	0.00064
2	TPI1	Up	3.75	0.00047
3	PRDX6	Up	3.6	0.0041
4	ECHS1	Up	3.31	0.0007
5	ARHGDIA	Up	2.89	0.00096
6	NME2	Up	2.13	0.0025
7	FGA	Up	1.98	0.0045
8	LMNA	UP	1.59	0.046
9	MB	Down	-1.5	0.021
10	MYL9	Down	-1.92	0.0032

**Table 3:** Clinicopathological features of early stage OTSCC (with follow-up after treatment) analyzed based on the vimentin protein expression.

Clinical parameters	*N* 150	Vimentin negative (*n* = 92)	Vimentin positive (*n* = 58)	*p* value
Age				
< 55 years	82	45 (54.9)	37 (45.1)	
> 55 years	68	47 (69.1)	21 (30.9)	
*Sex*				
Male	102	65 (63.7)	37 (36.3)	
Female	48	27 (56.2)	21 (43.8)	
Site				
Lateral border	132	77 (58.3)	55 (41.7)	
Tip	2	0	2 (100)	*p* = 0.012
Dorsum	5	5 (100)	0	
Ventral aspect	11	10 (90.9)	1 (9.1)	
Stage				
Stage 1	56	42 (75)	14 (25)	*p* = 0.008
Stage 2	94	50 (53.2)	44 (46.8)	
Tumor size				
0-2 cm	58	43 (74.1)	15 (25.9)	
2.1-3 cm	89	46 (51.7)	43 (48.3)	*p* = 0.009
> 3 cm	3	3 (100)	0	
Pattern				
Exophytic	42	22 (52.4)	20 (47.6)	
Infiltrating	88	52 (59.1)	36 (40.9)	*p* = 0.014
Ulcerated	20	18 (90)	2 (10)	
Grade				
WDSCC	116	73 (62.9)	43 (37.1)	
Mod to poorly	27	14 (51.8)	13 (48.1)	
Tobacco habits				
Chewer	44	21 (47.7)	23 (52.3)	
Smoker	27	19 (70.4)	8 (29.6)	
Chewer + smoker	17	12 (70.6)	5 (29.4)	
Non user	62	40 (64.5)	22 (35.5)	
Alcohol (yes)	21	13 (61.9)	8 (38.1)	
Upfront management				
Observation	73	51 (69.9)	22 (30.1)	
RND	33	17 (51.5)	16 (48.5)	
Rad to neck	24	24 (54.5)	20 (45.5)	
Failure pattern				
No evidence of disease	65	46 (70.8)	19 (29.2)	
Local recurrence	27	14 (51.9)	13 (48.1)	*p* = 0.028
Nodal recurrence	18	14 (77.8)	4 (22.2)	
Locoregional recurrence	39	18 (46.2)	21 (53.8)	
Distant metastasis	1	0	1 (100)	
Treatment outcome				
No evidence of disease (NED)	65	46 (70.8)	19 (29.2)	*p* = 0.038
Failure	85	46 (54.1)	39 (45.9)	
Survival				
Alive-NED	84	49 (58.3)	34 (40.4)	
Alive with disease	4	4 (100)	—	
Dead	63	39 (61.9)	24 (38.1)	

**Table 4:** Vimentin expression at invasive tumor front (ITF) vs. pattern of failure in early staged OTSCC with treatment follow-up.

Vimentin status	No evidence of disease (NED)	Local recurrence	Nodal recurrence	Locoregional recurrence
Negative expression at ITF (*n* = 55)	28 (50.9)	9 (16.4)	10 (18.2)	8 (32)
Positive expression at ITF (*n* = 50)	20 (40)	10 (20)	3 (16)	17 (68)

*p* = 0.002; *χ*^2^ = 14.792.

**Table 5:** Vimentin expression in oral precancers and oral buccal cancers along with normal.

Clinical parameters	*N* 195	Vimentin negative (*n* = 144)	Vimentin positive (*n* = 51)	*p* value
Age				
< 45 years	93	81 (87.1)	12 (12.9)	*p* ≤ 0.001
> 45 years	102	63 (61.8)	39 (38.2)	*χ* ^2^ = 16.163
*Sex*				
Male	139	107 (77)	32 (23)	
Female	56	37 (66.1)	19 (33.9)	
Diagnosis				
Normal	42	42 (100)	0	
Mild dysplasia	24	22 (91.7)	2 (8.3)	
Moderate dysplasia	13	10 (76.9)	3 (23.1)	
Severe dysplasia	13	11 (84.6)	2 (5.4)	*p* ≤ 0.001
OSMF	32	31 (96.8)	1 (3.2)	*χ* ^2^ = 77.037
WDSCC	40	17 (42.5)	23 (57.5)	
MDSCC	29	9 (31)	20 (69)	
Verrucous	3	3 (100)	0	
Habits				
Pan	96	70 (72.9)	26 (27.1)	
Betel quid	35	21 (60)	14 (40)	*p* = 0.045
Sharp tooth	64	53 (82.8)	11 (17.2)	*χ* ^2^ = 6.181

## Data Availability

The complete proteomic profiling data used to support the findings of this study have not been made available at the moment due to the patent and project grant (BIRAC PACE) submissions based on the data.
